# Clinical Use of HIV Integrase Inhibitors: A Systematic Review and Meta-Analysis

**DOI:** 10.1371/journal.pone.0052562

**Published:** 2013-01-09

**Authors:** Peter Messiaen, Annemarie M. J. Wensing, Axel Fun, Monique Nijhuis, Nele Brusselaers, Linos Vandekerckhove

**Affiliations:** 1 General Internal Medicine and Infectious Diseases, Ghent University Hospital, Ghent, Belgium; 2 Virology, Department of Microbiology, University Medical Center Utrecht, Utrecht, The Netherlands; Centro de Biología Molecular Severo Ochoa (CSIC-UAM), Spain

## Abstract

**Background:**

Optimal regimen choice of antiretroviral therapy is essential to achieve long-term clinical success. Integrase inhibitors have swiftly been adopted as part of current antiretroviral regimens. The purpose of this study was to review the evidence for integrase inhibitor use in clinical settings.

**Methods:**

MEDLINE and Web-of-Science were screened from April 2006 until November 2012, as were hand-searched scientific meeting proceedings. Multiple reviewers independently screened 1323 citations in duplicate to identify randomized controlled trials, nonrandomized controlled trials and cohort studies on integrase inhibitor use in clinical practice. Independent, duplicate data extraction and quality assessment were conducted.

**Results:**

48 unique studies were included on the use of integrase inhibitors in antiretroviral therapy-naive patients and treatment-experienced patients with either virological failure or switching to integrase inhibitors while virologically suppressed. On the selected studies with comparable outcome measures and indication (n = 16), a meta-analysis was performed based on modified intention-to-treat (mITT), on-treatment (OT) and as-treated (AT) virological outcome data. In therapy-naive patients, favorable odds ratios (OR) for integrase inhibitor-based regimens were observed, (mITT OR 0.71, 95% CI 0.59–0.86). However, integrase inhibitors combined with protease inhibitors only did not result in a significant better virological outcome. Evidence further supported integrase inhibitor use following virological failure (mITT OR 0.27; 95% CI 0.11–0.66), but switching to integrase inhibitors from a high genetic barrier drug during successful treatment was not supported (mITT OR 1.43; 95% CI 0.89–2.31). Integrase inhibitor-based regimens result in similar immunological responses compared to other regimens. A low genetic barrier to drug-resistance development was observed for raltegravir and elvitegravir, but not for dolutegravir.

**Conclusion:**

In first-line therapy, integrase inhibitors are superior to other regimens. Integrase inhibitor use after virological failure is supported as well by the meta-analysis. Careful use is however warranted when replacing a high genetic barrier drug in treatment-experienced patients switching successful treatment.

## Introduction

Since the first reports on Acquired Immunodeficiency Syndrome (AIDS), the human immunodeficiency virus (HIV) has caused a devastating pandemic with yearly 2.6 million new infections worldwide [1]. The stable integration of the reverse transcribed viral genome into host chromatin forms an important point-of-no-return during HIV infection. Raltegravir is the first representative of a new class of antiretroviral drugs targeting the strand transfer reaction during this integration process. Strand transfer integrase inhibitors bind in the catalytic core domain of the enzyme and compete for binding with host DNA. Introduction of raltegravir in 2008 appeared almost simultaneously with approval of second generation drugs of existing therapeutic classes as the protease inhibitor (PI) darunavir and the non-nucleoside reverse transcriptase inhibitor (NNRTI) etravirine. Combined use of these drugs has resulted in high levels of virological suppression in treatment-experienced populations [2], [3]. As a result, the treatment goals in highly experienced patients have been redefined towards successful suppression of plasma viral load [4], [5]. In addition to high efficacy, the initial use of this first integrase inhibitor (INI) also suggested good tolerability, a favorable safety profile and absence of significant drug-drug interactions. Following this success, raltegravir has been explored in a divergent setting of clinical indications such as therapy-naive populations, once-daily formulations, simplification strategies, nucleoside/nucleotide reverse transcriptase inhibitors sparing regimens and maintenance therapy. Conflicting results were reported in several clinical situations, hampering uniform conclusions for successful use of raltegravir. Meanwhile other INIs with a similar mechanism of action such as elvitegravir and dolutegravir have been clinically evaluated. Elvitegravir has been approved in the US and dolutegravir has entered advanced stages of clinical development (Table 1). The objective of this study was to perform a systematic review and meta-analysis of current evidence regarding the use of integrase inhibitors in various clinical settings.

**Table 1:** Main characteristics of the integrase inhibitors used in clinical practice or in clinical trials in humans.

Generic Name	FDA/EMA status	Dosing Recommendations	Serum Half-life	Route of Metabolisation	Major Adverse Events
Raltegravir	FDA/EMA approved for therapy-naive and experienced patients	400 mg BD - no food restrictions	∼9 hrs	UGT1A1- mediated glucuronidation	nausea and diarrhea - skin rash with fever (rare) - CPK elevation, muscle weakness, rhabdomyolysis - transient elevation of serum transaminase levels - hypersensitivity reactions, hepatitis
Elvitegravir	FDA approved for therapy-naive patients as part of single tablet regimen	150 mg QD, + booster (100 mg ritonavir or cobicistat) - to be taken with meals	∼9,5 hrs if boosted	Predominantly cytochrome P450 (CYP3A4) metabolized, minor pathways via UGT1A1/3 glucuronidation and oxidative metabolism	nausea and diarrhea - headache, insomnia - eGFR decrease when combined with cobicistat (inhibition of tubular secretion of creatinine)
Dolutegravir	Phase III studies ongoing	50 mg QD in INI- naive patients, 50 mg BD in INI-experienced patients - no food restrictions	∼15 hrs	Predominantly UGT1A1- mediated glucuronidation, cytochrome P450 (CYP3A4) metabolisation as minor pathway	nausea and diarrhea - transient low-level increases in serum creatinine - eGFR decrease (inhibition of tubular secretion of creatinine) - hypersensitivity reactions, hepatitis

FDA (Food and Drug Administration) and EMA (European Medicines Agency) status , dosing recommendations, serum-half-life, main route of metabolization and currently reported major adverse events are indicated.

BD = twice-daily; QD = once-daily.

## Methods

### Data Sources and Searches

We followed a protocol using the methodological approaches outlined in the Agency for Healthcare Research and Quality Methods Guide for Effectiveness and Comparative Effectiveness Reviews [6] and applied the PRISMA Guidelines [7]. The systematic literature review aimed at including all published studies from April 2006 until November 2012 reporting on the clinical use of INIs for antiretroviral therapy. We searched MEDLINE and Web-of-Science with the MeSH terms “integrase inhibitor”, “HIV” or “raltegravir” or “elvitegravir” or “dolutegravir”. We systematically hand-searched the meeting proceedings (abstract books, trial registries and reference lists) from key conferences that were held in the same period: the Conference on Retroviruses and Opportunistic Infections, the European Workshop on HIV & Hepatitis: Treatment Strategies & Antiviral Drug Resistance, the International HIV Drug Resistance Workshop, the International AIDS Conference, the European AIDS Conference (EACS), the International Congress on Drug Therapy in HIV Infection and the Interscience Conference on Antimicrobial Agents and Chemotherapy.

### Study Selection

**Figure 1:**
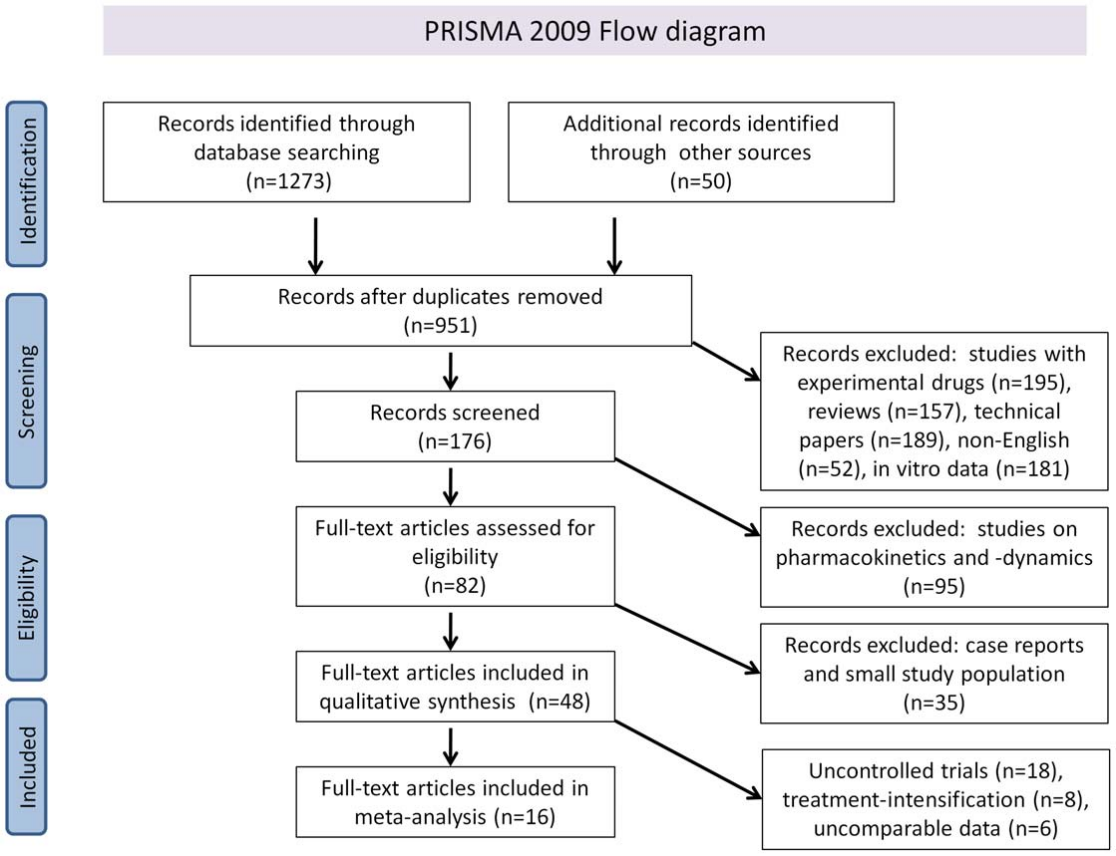
Prisma 2009 Flow diagram literature search and study selection. PRISMA diagram showing the different steps of systematic review, starting from literature search to study selection and exclusion. At each step, the reasons for exclusion are indicated.

The initial selection was performed by two independent investigators. We included original research papers or abstracts of clinical trials on the use of INIs in HIV-positive patients. We included randomized controlled trials, non-randomized trials, retrospective analysis of these trials, cohort studies or cross-sectional studies. Language restrictions were set on English. We excluded *in vitro* and animal studies, review articles, studies with experimental drugs currently not evaluated in clinical trials in humans, studies on the prophylactic use of INIs and studies in pediatric patient populations (younger than 16 years). We assessed all titles and abstracts identified by our search and excluded reviews or reports describing obviously different topics other than clinical data related to INI use. Discrepancies were resolved by consensus or by consulting a third reviewer. Of the remaining reports, we read the abstracts and excluded reports if they dealt with non-clinical factors or described only pharmacokinetic and pharmacodynamic data. Case reports and studies with small patient cohorts (n<10) were excluded and subsequently full-length articles were retrieved from all published papers. The flow diagram is depicted in Figure 1.

### Data Extraction and Quality Assessment

**Figure 2:**
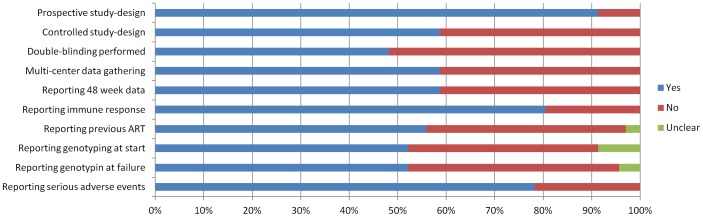
Quality assessment of the selected studies in systematic review. Summary of the proportion of studies that fulfilled each quality assessment criterion. When no clear answer could be obtained for a specific criterion, it was classified as “unclear”. ART = Antiretroviral Treatment.

All selected articles or abstract-only reports were carefully read and analyzed. The quality assessment of the studies selected in the systematic review is depicted in Figure 2. We assessed the strength of evidence by using the GRADE [Grading of Recommendations Assessment, Development and Evaluation] approach [8] (Table 2). In this way, a body of evidence is evaluated regarding four major domains: risk of bias, consistency, directness and precision of study outcomes. This results in four strength of evidence grades: high, moderate, low or insufficient.

**Table 2:** Overview of studies in systematic review, grouped according to study-design and indication: regimens, population size, treatment duration and summary of main outcome data and conclusions.

	INI (n = )	CTRL (n = )	Regimen	(w)	Summary
**ART-naive patients: INI in combination with dual NRTI**					**GRADE: HIGH**
**STARTMRK ** [**15**]–[**19**]	281	282	RAL 400 mg bd + TDF/FTC vs. EFV + TDF/FTC	240	Non-inferiority of raltegravir in reaching VL<50 c/ml (71% vs 61.3% EFV, mITT); Significantly more rapid decline of viral load in early phase with INI; Mean CD4 increase 374 (INI) versus 312 cells/ml (EFV).
**Protocol 004 ** [**20**]–[**22**]	160	38	RAL 100, 200, 400 or 600 mg bd + TDF/3TC vs. EFV + TDF/3TC	240	Similar proportions of VL<50 c/ml (69% vs 63%, mITT) in all dosages (400 mg bd single arm as from w48) - non-inferiority for raltegravir; Similar mean CD4 increase (302 versus 267 cells/ml); Less frequent drug-related clinical adverse events with raltegravir (55%) than efavirenz (76%).
**SHIELD ** [**24**]	35	N	RAL 400 mg bd + ABC/3TC	96	Proportion of VL<50 c/ml (mITT: 77%); Median CD4 increase 304 cells/ml; No drug-related serious adverse events reported
**GS-236-014 ** [**26**]	48	23	EVG/COBI single tablet qd+ TDF/FTC vs. EFV + TDF/FTC	48	Non-inferiority of elvitegravir/cobicistat in suppressing VL<50 c/ml (mITT: 90% vs 83% treatment difference +8.4% (−8.8 to +25.6%). Treatment with EVG/COBI associated with more rapid achievement of undetectable VL than EFV/FTC/TDF (P<0.05 at weeks 2,4 and 8). Lower rate of drug-related central nervous system and psychiatric adverse events in EVG/COBI group
**GS-236-0102 ** [**25**]	348	352	EVG/COBI/FTC/TDF qd vs EFV/TDF/FTC	48	Non-inferiority of QUAD (EVG/COBI/TDF/FTC) in suppressing VL<50 c/ml (mITT 87.6% vs 84.1%; treatment difference +3.6% CI −1.6 to +8.8%). Treatment with QUAD associated with higher CD4 increase at 48w (239 cells/µL vs 206 cells/µL p = 0.009). Similar numbers of patients discontinued treatment because of an adverse event in each group. Nausea was more common in the QUAD group, CNS and psychiatric adverse events more frequent with EFV
**SPRING-1 ** [**27**]–[**29**]	155	50	DTG 10,25 or 50 mg + TDF/FTC or ABC/3TC vs. EFV + TDF/FTC or ABC/3TC	48	Similar response rates (VL<50 c/ml) for all doses of dolutegravir compared to efavirenz (mITT 87% versus 82%); Median CD4 increase in all dolutegravir groups were higher than efavirenz (231 cells per µL vs 174 cells per µL; p = 0·076); No serious adverse events related to dolutegravir
**SINGLE ** [**14**]	414	419	DTG 50 mg + ABC/3TC vs. EFV/TDF/FTC	48	Significant better virological reponse of DTG/ABC/3TC compared to EFV/TDF/FTC (mITT 88% vs 81%); median CD4 increase significantly higher in DTG/ABC/3TC (267 vs 208 cells/µl; p<0.001) No INI or NRTI resistance observed in the DTG-treated group and no serious adverse events.
**GS-236-0103 ** [**31**]	353	355	EVG/COBI/FTC/TDF qd vs ATV/r + TDF/FTC	48	Non-inferiority of QUAD (EVG/COBI/TDF/FTC) in suppressing VL<50 c/ml (mITT 89.5% vs 86.8%; treatment difference +3.0% CI −1.9 to +7.8%). Similar increase of CD4 count in both groups. Similar numbers of patients discontinued treatment because of an adverse event in each group. More Grade 3 and 4 lab abnormalities in the ATV/r group compared to QUAD.
**SPRING -2 ** [**30**]	413	414	DTG 50 mg qd + TDF/FTC or ABC/3TC vs RAL 400 mg bd + TDF/FTC or ABC/3TC	48	Non-inferiority of dolutegravir versus raltegravir in reaching VL<50 c/ml (ITT 88% vs 85%). Similar median CD4 increase (230 CD4 cells/µl). Discontinuation due to serious adverse events 2% in each group. No IN or NRTI resistance upon failure in DTG group versus 1 and 4 pts in the RAL groups.
**QDMRK ** [**23**]	382	388	RAL 800 mg bd + TDF/FTC vs. RAL 400 mg bd + TDF/FTC	48	mITT: 83% in the once-daily group had virological response compared with 89% in the twice-daily group (difference −5·7%, 95% CI −10·7 to −0·83; p = 0·044); Mean CD4+ increase comparable in both groups; serious adverse events reported in 7% and 10% of resp. once-daily recipients and twice-daily recipients.
**ART-naive patients: INI in combination with PI**					**GRADE: MODERATE**
**SPARTAN ** [**13**]	63	31	RAL 400 mg bd + ATV vs. ATV/r + TDF/FTC	24	Through week 24, both arms achieved comparable efficacy rates (ITT 74,6% versus 63,3% VL<50 c/ml)
**PROGRESS ** [**32**]	101	105	RAL 400 mg bd + LPV/r vs. LPV/r + TDF/FTC	48	Non-inferiority of the study regimen at reaching VL<40 c/ml at week 48 (ITT 81.2% versus 85.7% ; difference −4.5%; 95% CI, −15.1% to 5.9%); Mean CD4 increase was similar between groups.
**ACTG A5262 ** [**34**]	112	N	RAL 400 mg bd + DRV/r	48	DRV/r plus RAL was effective (mITT 73% VL<50 c/ml at week 48) and well tolerated in treatment-naive patients, but those with base-line viral load >100,000 copies/mL had more VF and INI resistance.
**RADAR ** [**33**]	40	40	RAL 400 mg bd + DRV/r vs. DRV/r + TDF/FTC	24	mITT VL<50 c/ml at week 24 achieved in 75.0% (RAL treated) versus 82.5%; mean CD4 increase +143 versus 109 cells/ml
**Fallon et al ** [**83**]	15	15	RAL 400 mg bd (naive) + LPV/r vs RAL 400 mg bd (exp) + LPV/r	48	mITT: 80.0% (12/15 treatment-naive) versus 73.3% (11/15 treatment-experienced) had VL<50 c/ml at 48 weeks; mean CD4 change 102 versus 66 cells/ml
**ART-experienced patients: virological failure**					**GRADE: MODERATE**
**BENCHMRK 1 and 2 ** [**2**], [**35**]	462	237	RAL 400 mg bd + NNRTI + NRTI vs. Placebo + NNRTI + NRTI	96	Sustained VL<50 c/ml in the combined studies of 57% (raltegravir) versus 26% (placebo) (mITT 96w); mean CD4 increase 109 versus 45 cells/ml (P<0.001 for each study individually and the combined studies); Frequencies and exposure-adjusted rates of clinical adverse events and laboratory abnormalities similar in both groups
**Protocol 005 ** [**36**], [**37**]	133	45	RAL 200, 400 or 600 mg bd + optimized BR vs. Placebo + optimzed BR	96	Raltegravir in all doses superior than placebo in reaching undetectable VL at double-blind phase (till 24 weeks); No dose-dependent differentiation in the safety or antiviral activity of raltegravir; After 96weeks (RAL 400 mg bd >24w all groups) 55% and 48% reached VL<400 c/mL and VL<50 c/ml (mITT); There were few discontinuations of raltegravir (4%) due to adverse events.
**ANRS 139 TRIO ** [**3**]	103	N	RAL 400 mg bd + ETV + DRV/r	96	mITT: 86% VL<50 c/ml at 48w; median CD4 increase 108 cells/ml. Grade 3 or 4 clinical adverse events reported 14,6%, though only 1 patient discontinued the regimen because of an adverse event
**Canestri et al ** [**39**]	20	N	RAL 400 mg bd + ETV + optimized BR	24	mITT: 65% of patients reached VL<40 c/ml and 100% VL<400 c/ml; median CD4 increase +80cells/ml
**Nozza et al ** [**41**]	28	N	RAL 400 mg bd + MVC + ETV	48	mITT/OT: At week 48, 26/28 patients achieved VL<50 c/ml. The median CD4 increase was 267 cells/µL. No patient discontinued treatment.
**Caby et al ** [**42**]	67	N	RAL 400 mg bd + BR	48	At 48 weeks, 43/67 patients had complete (VL<50 c/ml) and 16/67 incomplete (VL<400 c/ml) suppression, while 8 patients failed (mITT). Upon failure, 6/8 patients harbored RAL resistance
**GS-183-105 ** [**38**]	205	73	EVG/RIT 20, 50 or 125 mg bd + optimized BR vs. PI/r + optimized BR	24	mITT: Elvitegravir 50 mg was noninferior and elvitegravir 125 mg superior compared with the PI/r (based on DAVG24 scores). Efficacy was impacted by activity of background agents. Similar mean CD4 increase across all treatment arms; no relationship between elvitegravir dosage and adverse events.
**GS-183-0145 ** [**43**]	361	363	EVG 150 mg qd + PI/r + NRTI vs. RAL 400 mg bd + PI/r + NRTI	48	Elvitegravir non-inferior (59%) compared to raltegravir (58%) in achieving complete virological response (mITT treatment diff erence +1·1%, 95% CI −6·0 to 8·2); Median CD4 increases and proportion of adverse events attributed to study drugs similar in the two treatment arms
**VIKING I ** [**45**], [**46**]	27	N	DTG 50 mg qd + optimized BR	24	mITT: 52% and 41% of patients treated till 24weeks achieved VL<400 c/ml and VL< 50 c/ml; Drug related AEs (any grade) were observed in 6 (22%) subjects
**VIKING II ** [**47**]	24	N	DTG 50 mg bd + BR	11d	After 11days of functional monotherapy (triple resistant virus including INI), 54% of patients reached VL<400 c/ml (mITT). No discontinuation due to AE/lab toxicities. 17% treatment emergent grade 3 lab abnormalities.
**ART-experienced patients: switch strategy**					**GRADE: LOW**
**SWITCHMRK 1 and 2 ** [**48**]	353	354	RAL 400 mg bd + BR − LPV/r vs. BR	24	84·4% in the raltegravir group versus 90·6% in the lopinavir-ritonavir group (mITT treatment diff erence −6·2%, −11·2 to −1·3) had VL<50 c/ml, leading to study stop. Majority of RAL-failures had RAL resistance. Mean CD4 increase was small and did not diff er between treatment groups.
**SPIRAL ** [**49**]	139	134	RAL 400 mg bd + BR − PI/r vs. BR	48	Non-inferiority of raltegravir (mITT 89.2% versus 86.6% of patients remained free of treatment failure [difference +2.6%; 95% CI −5.2 to +10.6]; No differences between treatment groups in CD4 increase
**EASIER ANRS 138 ** [**50**]	85	85	RAL 400 mg bd + BR − T20 vs. BR +− T20 or RAL (>24w)	48	At week 48, 90% of patients in both the immediate and deferred groups had plasma VL<50 c/ml (mITT); Median CD4 cell counts remained stable during follow-up.
**ODIS ** [**55**]	149	73	RAL 800 mg qd + BR − PI/r vs. RAL 400 mg bd + BR − PI/r	24	6.4% in the oncedaily arm and 2.9% in the twice-daily arm (mITT) experienced virological failure, with significant higher rates in patients with prior nucleoside reverse transcriptase inhibitor resistance (16,2% versus 0,7% P<0,001); significant increase in CD4 (+32 cells/µL) after switch to RAL.
**RASTA ** [**56**]	21	19	RAL 400 mg bd + TDF/FTV vs. RAL 400 mg bd + ABC/3TC	24	One virological failure in TDF/FTC arm at 24 weeks; At 24w, a higher increase in CD4 count was observed in arm B versus arm A (mean +62 vs −9 cells/mm3 respectively, p = 0.04).
**Talbot et al ** [**53**]	28	N	RAL 400 mg bd + BR − T20	24	26/27 patients with data at 24 weeks remained with a VL <50 c/ml; No significant changes, statistically or clinically, were observed in the CD4 counts
**CHEER ** [**54**]	52	N	RAL 400 mg bd + BR − T20	24	49/52 (94.2%, confidence interval: 1.2% to 15.9%) remained with a VL<50 c/ml 24 weeks (mITT); mean CD4 increase of 32 cells/ml was seen after 24 weeks
**Harris et al ** [**51**]	35	N	RAL 400 mg bd + BR − T20	16	34/35 patients have HIV RNA <50 c/ml at 16 weeks of follow-up (mITT)
**Santos et al ** [**52**]	36	N	RAL 400 mg bd + BR − T20	48	All but 1 patient (discontinuation) maintained VL<50 copies/mL at Weeks 24 and 48
**Reliquet et al ** [**57**]	20	N	RAL 400 mg bd + NVP	48	At week 48, 19/20 patients (100% undetectable VL at start) achieved VL<50 c/ml (mITT)
**ART-experienced patients: treatment intensification**					**GRADE: INSUFFICIENT**
**Hatano et al ** [**63**]	15	15	RAL 400 mg bd + BR vs. Placebo + BR	48	The proportion of subjects with undetectable VL did not differ between the 2 groups (mITT p = 0.42); Raltegravir intensification did not have a significant effect on immune activation or HIV-specific responses in PBMCs or gut-associated lymphoid tissue.
**CORAL ** [**59**]	19	54	RAL 400 mg bd + BR vs. HIBC/placebo + BR	24	Compared with placebo, the addition of neither raltegravir nor HIBC to cART for 24 weeks resulted in a significant change in CD4 count (mITT mean difference, 95% confidence interval [CI]: 3.09 cells/lL, 214.27; 20.45, p = .724 and 9.43 cells/lL, 27.81; 26.68, p = .279, respectively)
**ACTG A5244 ** [**62**]	25	24	RAL 400 mg bd + BR vs. placebo + BR	12	12 weeks of raltegravir intensification did not demonstrably reduce low-level plasma viremia in patients on currently recommended ART.
**Buzon et al ** [**58**]	45	24	RAL 400 mg bd + BR vs. BR	48	Raltegravir intensification of a three-drug suppressive ART regimen resulted in a specific and transient increase in episomal DNAs in a 29% of ART-suppressed subjects; With these episomal DNAs, immune activation was higher at baseline and was subsequently normalized after raltegravir intensification.
**McMahon et al ** [**65**]	10	N	RAL 400 mg bd + BR	4	There was no evidence in any subject of a decline in HIV-1 RNA level (ultra-sensitive assay) during the period of raltegravir intensification or of rebound after discontinuation
**Cesar et al ** [**60**]	10	10	RAL 400 mg bd + BR vs. Placebo + BR	48	After 48 weeks all patients remained with VL<5 c/mL. No differences in CD4 gain were observed between placebo and raltegravir arms (mITT 11 vs. −4 respectively, t18 = 0.586, p = 0.565); Increased immune activation did not change after 48 weeks
**Lichtenstein et al ** [**64**]	30	N	RAL 400 mg bd+ BR	12	Addition of raltegravir to a suppressive ART regimen improves some immunologic, cytokine/chemokine, and effector memory cell parameters (IFNγ, MIP-1α; IL-2 and RANTES) in immunologic non-responders.
**Dahl et al ** [**66**]	14	9	RAL 400 mg bd + BR vs BR	12	Raltegravir intensification did not reduce intrathecal immunoactivation or alter CSF HIV-1 RNA levels in subjects with baseline viral suppression
**ART-experienced patients: INI in combination with PI**					**GRADE: INSUFFICIENT**
**Ripamonti et al ** [**69**]	27	N	RAL 400 mg bd + ATV	24	After a median follow up of 7 (IQR 5–7) months, VL was <50 c/ml in all but one of the 27 patients (63% undetectable VL at start); The median CD4 count increment was 168 cells/µl.
**Tsukada et al ** [**70**]	19	N	RAL 400 mg bd + DRV/r	48	After a median follow up of 47 (24–102) weeks, VL<100 c/mL detected in 16/19 patients (68% undetectable VL at start)
**Allavena et al ** [**67**]	29	N	RAL 400 mg bd + PI/r	22	After a median follow-up of 22 weeks, VL<50 c/ml in 24/29 patients (79% undetectable VL at start)
**Cordery et al ** [**68**]	20	N	RAL 400 mg bd + ATV	72	At week 72, 13/20 patients (100% undetectable VL at start) achieved VL<50 c/ml (mITT) Median CD4 cell counts remained stable during follow-up.
**Gardner et al ** [**71**]	39	N	RAL 400 mg bd + PI	48	After median follow-up of 47 weeks, 74% and 44% of patients reached HIV RNA<200 c/ml and <50 c/ml – mITT (46% HIV RNA<200 c/ml at start) in heavily pre-treated patients. Adherence and pre-existing PI resistance are associated with virological failure.

GRADE level of evidence per category is added. INI-containing treatment arms are underlined.

(c)ART = (combination) antiretroviral treatment; INI = integrase inhibitor; CTR = control arm; (w) = weeks; VL<50 = viral load or HIV RNA <50 copies/ml; N = not applicable; RAL = raltegravir; EFV = efavirenz; EVG = elvitegravir; COBI = cobicistat; DTG = dolutegravir; ATV = atazanavir; DRV = darunavir; TDF/FTC = tenofovir/emtricitabine; ABC/3TC = abacavir/lamivudine; LPV = lopinavir; r = ritonavir; (N)NRTI = (non-)nucleoside reverse transcriptase inhibitor; PI = protease inhibitor; BR = background regimen; T20 = enfurvirtide; NVP = nevirapin; HIBC = hyperimmune bovine colostrum.

### Data Synthesis

The following data were collected: (a) basic study characteristics: study period, prospective or retrospectively gathered data, number of participating centers; (b) population characteristics: population size, pre-trial antiretroviral treatment, exclusion criteria; (c) intervention characteristics: drugs used, drug dosage, duration of treatment and follow-up; (d) outcome parameters: virologic and immunologic response, genotypic data of eventual drug resistance at therapy failure, clinical and laboratory adverse events.

### Data Analysis

A random-effects meta-analysis was used to investigate the combination or interaction of this collection of independent studies. This was performed using STATA/MP4 (release 11; StataCorp LP, Texas USA; STATA module ‘mais’) following the Mantel-Haenszel model to obtain weighted odds ratios (OR) and 95% confidence intervals (CI) of virological outcome data [9], [10]. An OR of one indicates no difference between both groups; ORs below one indicate benefit of INI versus the control regimen. If the 95% CI of the OR contains the value 1, there is no sufficient evidence for a difference between both treatment groups. For the calculation of these ORs, the virological outcome data were normalized towards time-point (24 or 48 weeks after start of INI). Studies reported virological outcome data based on: TLOVR (time-to-loss-of-virological-response) (n = 2), snapshot approach (n = 3) or protocol-defined composite-endpoints (n = 11). In order to reveal differences between virological efficacy and ancillary benefits (e.g. lower toxicity, more convenient formulation), we extracted modified intention-to-treat (mITT) as well as on-treatment (OT) and as-treated (AT) data [11], [12]. mITT includes all patients who received at least one dose of study drug and completed the study, missing data are considered as failures, as are non-completers. OT includes only the patients completing the study at the analyzed endpoint. Patient-data were censored in case of toxicity, loss to follow-up, lack of efficacy before the endpoint is reached and other reasons. AT is similar to OT, but includes patients with virological failure before the endpoint is reached. Only controlled studies with virological outcome data comparing INI versus another compound or placebo were included. If data were not available in the paper, authors were contacted and invited to provide it. We assessed statistical heterogeneity using the I-square statistic that measures the degree of inconsistency across studies; it results in a 0–100% range quantifying the proportion of variation in the effect, which is due to inter-study variation, with lower values indicating more homogenous study results. We predefined heterogeneity (I^2^≤25% for low, 25%<I^2^<50% for moderate, and I^2^≥50% for high). Funnel plots in different subcategories were constructed to assess bias. A pooled analysis was made of all available data on immunological efficacy, adverse events and emergence of drug resistance when using INIs.

## Results

### Systematic review

The systematic review resulted in 48 eligible studies on the clinical use of integrase inhibitors, of which 15 abstract-only reports (Figure 1). These studies include in total more than 9400 HIV-infected patients. Of these studies, 38 (79%) described interventions regarding raltegravir use. Elvitegravir and dolutegravir were respectively investigated in 5 (10%) studies each. The average study population size was 202 (IQR 28–222), the average study duration 48 weeks (IQR 24–48). All but four of the included studies were prospective, the majority randomized (59%) and multi-centered (59%). Blinding was performed in 48% of the studies, 20 studies were single-armed (Figure 2). Study characteristics of all studies with latest result updates and evidence levels per category can be found in Table 2, the studies and data used in the meta-analysis are listed in Table 3.

**Table 3:** Study characteristics of studies included in meta-analysis (n = 16): regimens, population size, timepoint of analysis and virological outcome data are enlisted.

	INI (n = )	CTR (n = )	Regimen	Analysis time point (w)
**ART-naive patients**				
STARTMRK [16]	281	282	RAL 400 mg bd + TDF/FTC vs. EFV + TDF/FTC	48
Protocol 004 [21]	160	38	RAL 100, 200, 400 or 600 mg bd + TDF/3TC vs. EFV + TDF/3TC	48
GS-236-014 [26]	48	23	EVG/COBI single tablet qd+ TDF/FTC vs. EFV + TDF/FTC	48
GS-236-0102 [25]	348	352	EVG/COBI/FTC/TDF qd vs EFV/TDF/FTC	48
SPRING-1 [28]	155	50	DTG 10,25 or 50 mg + TDF/FTC or ABC/3TC vs. EFV + TDF/FTC or ABC/3TC	48
SINGLE [14]	414	419	DTG 50 mg + ABC/3TC vs. EFV/TDF/FTC	48
GS-236-0103 [31]	353	355	EVG/COBI/FTC/TDF qd vs ATV/r + TDF/FTC	48
SPARTAN [13]	63	31	RAL 400 mg bd + ATV vs. ATV/r + TDF/FTC	24
PROGRESS [32]	101	105	RAL 400 mg bd + LPV/r vs. LPV/r + TDF/FTC	24
RADAR [33]	40	40	RAL 400 mg bd + DRV/r vs. DRV/r + TDF/FTC	24
**ART-experienced patients with virological failure**				
BENCHMRK 1 and 2 [2]	461	237	RAL 400 mg bd + NNRTI + NRTI vs. Placebo + NNRTI + NRTI	24
Protocol 005 [36], [37]	134	45	RAL 200, 400 or 600 mg bd + optimized BR vs. placebo + optimized BR	24
GS-183-105 [38]	205	73	EVG/RIT 20, 50 or 125 mg bd + optimized BR vs. PI/r + optimized BR	24
**ART-experienced patients switching suppressive therapy**				
SWITCHMRK 1 and 2 [48]	353	354	RAL 400 mg bd + BR − LPV/r vs. BR	24
SPIRAL [49]	139	134	RAL 400 mg bd + BR − PI/r vs. BR	32
EASIER ANRS 138 [50]	85	85	RAL 400 mg bd + BR − T20 vs. BR +− T20 or RAL (>24w)	24

INI-containing treatment arm is underlined.

ART = antiretroviral treatment; INI = integrase inhibitor; CTR = control arm; VL<50 = viral load or HIV RNA <50 copies/ml; RAL = raltegravir; EFV = efavirenz; EVG = elvitegravir; COBI = cobicistat; DTG = dolutegravir; ATV = atazanavir; DRV = darunavir; TDF/FTC = tenofovir/emtricitabine; ABC/3TC = abacavir/lamivudine; LPV = lopinavir; r = ritonavir; (N)NRTI = (non-)nucleoside reverse transcriptase inhibitor; PI = protease inhibitor; BR = background regimen; T20 = enfurvirtide.

### Meta-analysis

**Figure 3:**
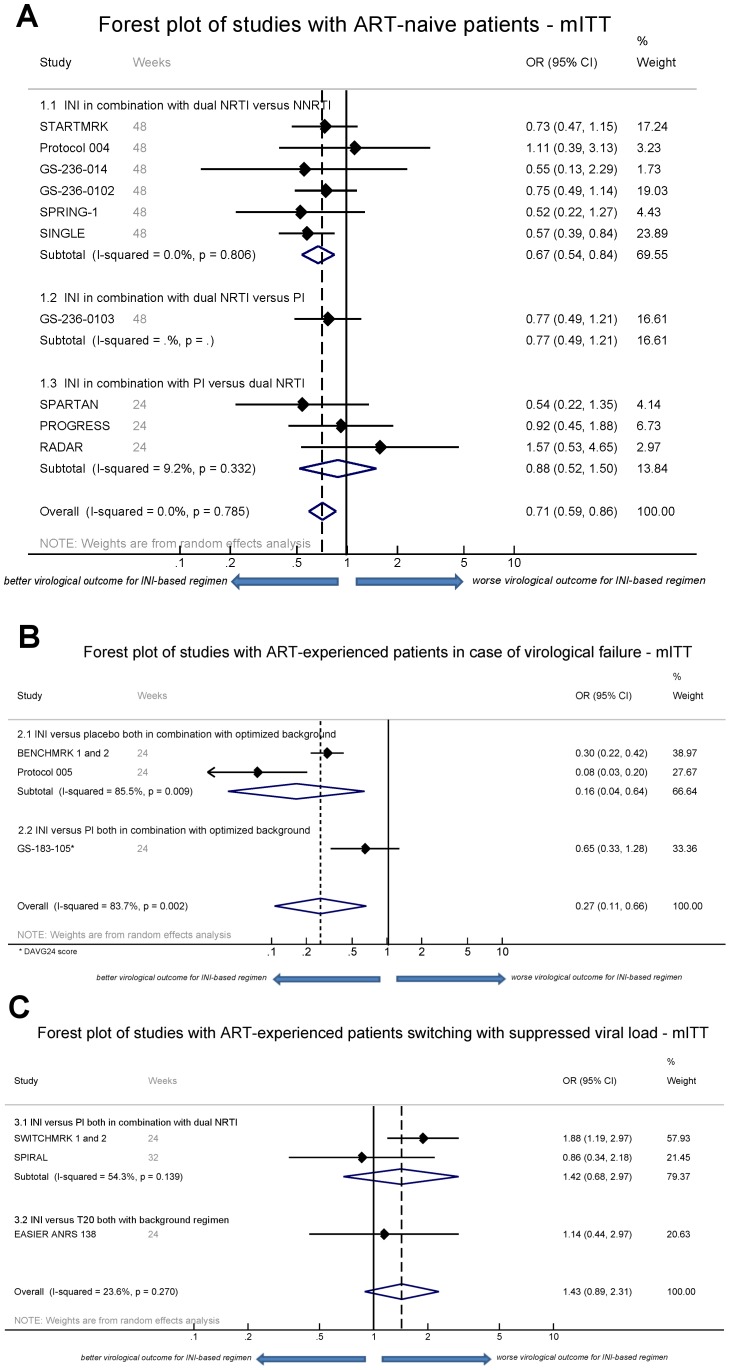
Forest Plot of mITT meta-analyses. Panel A: Forest plot showing the meta-analysis of mITT data extracted from studies with therapy-naïve patients. Besides an overall analysis, three sub-analyses for three different comparisons are depicted. The black line indicates OR = 1, signifying no benefit of the INI arm compared to the non-INI arm. The dotted line shows the odds ratio of all included studies. The individual odds ratios as well as the proportionate weight in the overall analysis are shown in the right column. Panel B: Forest plot showing the meta-analysis of mITT data extracted from studies with ART-experienced patients in case of virological failure. Panel C: Forest plot showing the meta-analysis of mITT data extracted from studies with ART-experienced patients switching with suppressed viral load. mITT = modified intention-to-treat; ART = antiretroviral treatment; INI = integrase inhibitor; (N)NRTI = (non-)nucleoside reverse transcriptase inhibitor; PI = protease inhibitor; T20 = enfuvirtide: OR = odds ratio.

Subsequently a meta-analysis of virological outcome (number of patients achieving HIV RNA below 50 copies/ml) was performed on the 16 controlled studies that compared an INI-based regimen with placebo or other drug classes for similar indications and in which similar endpoints could be evaluated (same measures and same available time-point results). This resulted in three subcategories (treatment-naive patients, treatment-experienced patients with virological failure and patients switching successful suppressive therapy) and the exclusion of studies on treatment intensification, due to the absence of comparable endpoints. The results of the meta-analysis are visualized in Forest plots (Figure 3 and Figure S1). Low heterogeneity in the outcome was seen in the treatment-naive subgroup (mITT, I^2^ 0.0%) and the patients switching successful suppressive therapy group (mITT, I^2^ 23.6). Higher heterogeneity was seen in the studies for patients experiencing virological failure (mITT, I^2^ 83.7%), which points to a higher inter-study variation on virological outcome (Figure S2).

### Clinical outcome in antiretroviral-naive patients

Based on our pre-defined criteria for meta-analysis, we included ten studies on treatment naïve patients. Overall, INI based regimens showed a better virological outcome, which reached significance in the mITT analysis (OR 0.71, 95% CI 0.59–0.86; Figure 3A) and OT analysis (OR 0.63, 95% CI 0.47–0.84; Figure S1A). The meta-analysis using AT data (OR 0.86, 95% CI 0.61–1.22; Figure S1A) showed a similar but non-significant favourable trend for INI-based regimens. For one study, no OT or AT data could be obtained [13], for another study AT data were lacking [14].

#### Comparison of INI versus NNRTI both in combination with dual NRTI

A sub-analysis of the virological outcome data at 48 weeks comparing INI versus NNRTI showed an OR favoring INIs over efavirenz in the mITT meta-analysis (OR 0.67, 95% CI 0.54–0.84) and OT meta-analysis (OR 0.59, 95% CI 0.43–0.81).

In STARTMRK, raltegravir twice-daily (n = 281) was compared to once-daily efavirenz (n = 282) with a backbone of tenofovir/emtricitabine [15]–[19]. Raltegravir showed non-inferiority based on the primary virological endpoint from 48 up to 240 weeks (mITT 48 week treatment difference +4.2%, 95% CI −1.9 to 10.3). Moreover viral decline in the early treatment phase was significantly more rapid in the raltegravir arm. In the rare cases resistance was observed, multiple raltegravir resistance associated mutations were detected (Table S1). In Protocol 004, an initial dose-ranging trial comparing raltegravir (n = 160) to efavirenz with tenofovir/lamivudine (n = 38) as backbone, similar virological and immunological results at 48 weeks (mITT) were observed as in STARTMRK at all doses [20]–[22]. Few but high-level raltegravir resistance was detected.

Amongst the studies with raltegravir in antiretroviral-naive patients which could not be incorporated in the meta-analysis, QDMRK, comparing once-daily raltegravir (800 mg qd) versus twice-daily raltegravir (400 mg bd), yields important additional information. Despite high levels of suppression in both arms, the once-daily arm was inferior compared to the twice-daily arm (mITT) [23]. This higher virological failure rate was observed mainly in patients starting with high baseline viral load and low C-through levels at 24 hours. Resistance was rare but more frequent in the once-daily arm. Also not included was the uncontrolled SHIELD study, which evaluated raltegravir in combination with abacavir/lamivudine (n = 35) and reported a high proportion (77%) of patients reaching undetectable viral load at 96 weeks in mITT analysis [24].

In the GS-236-0102 phase 3 study, elvitegravir combined with the booster cobicistat and a backbone of emtricitabine and tenofovir (QUAD) (n = 348) was compared to efavirenz with the same backbone (n = 352) both formulated as single tablet regimens (STR). The QUAD STR showed non-inferiority based on the primary virological endpoints up to 48 weeks (mITT 48 weeks treatment difference: +3.6% CI −1.6 to +8.8%) [25]. As has been reported for studies with raltegravir, a more rapid initial HIV RNA decline with elvitegravir was observed compared to the efavirenz arm. In both arms, similar small proportions of patients developed drug resistance upon therapy failure (both arms n = 8). In case of INI resistance in the QUAD failure group, NRTI resistance was observed as well, while in the comparator arm the detection of resistance was mainly limited to NNRTIs.

In the smaller GS-236-014 phase 2 study, the elvitegravir containing QUAD STR (n = 48) was also compared to an efavirenz containing STR (n = 23) with the same NRTI-backbone [26]. Although more patients in the elvitegravir arm achieved an undetectable viral load after one year of follow-up (mITT +8.4%, 95% CI −8.8 to +25.6), this was not statistically significant. Treatment failures were rare and no drug resistance could be assessed. The INI based regimen was well tolerated and fewer adverse events were reported.

In SPRING-1, a phase II dose-ranging randomized trial, a third INI dolutegravir was evaluated. Three different once-daily dosing arms (n = 51 each) were tested against efavirenz (n = 50) with either abacavir/lamivudine or tenofovir/emtricitabine [27]–[29]. Interim results at 48 weeks of follow-up provide favorable virological outcome in all dolutegravir arms driven by better tolerability (mITT, % of patients with HIV RNA <50 copies/ml: 87% dolutegravir arm versus 82% efavirenz arm). In the few cases of treatment failure, the interim analysis at 48 weeks from SPRING-1 did not detect mutations associated with dolutegravir resistance.

The follow-up study SPRING-2 compared dolutegravir 50 mg (n = 413) versus raltegravir 400 mg (n = 414), both combined with dual NRTI backbone [30]. This study was not included in the meta-analysis since it compared two INIs. After 48 weeks, similar proportions of patients on both INI regimens achieved undetectable viremia (ITT, % of patients with HIV RNA <50 copies/ml: 88% versus 85%). In the dolutegravir treated group, no resistance was detected upon failure, while in the raltegravir treated patients one INI and four NRTI mutations were observed.

The SINGLE trial compared two STR in therapy-naïve patients: dolutegravir combined with abacavir/lamivudine (n = 414) versus efavirenz combined with tenofovir/emtricitabine (n = 419) [14]. A significantly better virological outcome after 48 weeks for the dolutegravir-treated group was reported (mITT treatment difference +7.4%; 95% CI +2.5 to +12.3; p = 0,003), while also a significant better immunological response (CD4 increase 267 cells/µl versus 204 cells/µl). The dolutegravir STR was very well tolerated and no INI or NRTI resistance was detected.

#### Comparison of INI versus PI both in combination with dual NRTI

In the GS-236-0103 study, the elvitegravir containing QUAD STR (n = 353) was evaluated against ritonavir boosted atazanavir combined with emtricitabine/tenofovir (n = 355) [31]. At 48 weeks, the QUAD regimen showed non-inferiority based on suppression below 50 copies/ml (mITT treatment difference +3.0% CI −1.9 to +7.8%). In five cases in the QUAD group, resistance was observed versus none in the boosted PI treated group. Of those five, primary INI resistance was seen in four and NRTI resistance in three patients.

#### Comparison of INI versus dual NRTI both in combination with PI

In the search for simplification strategies, INI based NRTI-sparing regimens have been explored. A sub-analysis of this simplification approach indicated a favorable but non-significant OR in favor of INI compared to 2 NRTI when both are combined with a PI at 24 weeks using mITT data (OR 0.88, 95% CI 0.52–1.50) (Figure 3A).

In the randomized open-label SPARTAN trial, raltegravir with unboosted atazanavir (n = 63) was compared to ritonavir boosted atazanavir plus tenofovir/emtricitabine (n = 31) [13]. Through week 24, both arms achieved comparable efficacy rates (ITT % of patients with HIV RNA <50 copies/ml: 74.6% versus 63.3%). The higher rates of hyperbilirubinemia with twice-daily atazanavir and an increased development of raltegravir resistance in the INI-treated group, prompted early termination of the study. Upon virological failure, four out of five evaluable raltegravir treated patients developed high-level raltegravir resistance. In the PROGRESS trial, raltegravir with ritonavir boosted lopinavir (n = 101) was compared to ritonavir boosted lopinavir (n = 105) with tenofovir/emtricitabine [32] and reported non-inferiority of the study regimen at reaching HIV RNA <40 copies/ml at week 48 (ITT, 81.2% versus 85.7%; difference −4.5%; 95% CI, −15.1% to 5.9%). Upon virological failure, two out of four evaluable raltegravir treated patients developed high-level raltegravir resistance.

The randomized open-label RADAR trial, where raltegravir with ritonavir boosted darunavir (n = 40) was compared with ritonavir boosted darunavir and tenofovir/emtricitabine (n = 40), reported inferior virological outcome for the raltegravir containing regimen after 24 weeks (mITT 75% versus 82.5% of patients with HIV RNA <50 copies/ml) [33].

The ACTG A5262 trial evaluating raltegravir with ritonavir boosted darunavir (n = 112) could not be included in the meta-analysis due to lack of a control arm [34]. Unexpected high levels of virological failure were observed at 48 weeks (mITT 26% or 28 on 112 subjects). Virologic failure and the emergence of INI resistance upon failure was associated with a baseline viral load >100.000 copies/ml. Of note, in this trial a high percentage of patients harbored NRTI resistance mutations in their viral population at baseline, which may serve as an indicator of undisclosed treatment experience or more extensive archived transmitted resistance than detected using regular *pol* resistance tests.

### Clinical outcome in treatment-experienced patients with virological failure

Data from three studies on ART-experienced patients with virological failure could be incorporated in the meta-analysis. Overall, a statistical significant OR in favour of INI use in this population was observed in the mITT meta-analysis (OR 0.27; 95% CI 0.11–0.66) (Figure 3B). The meta-analyses using OT data (OR 0.28, 95% CI 0.20–0.38) and AT data (OR 0.16, 95% CI 0.04–0.61) included two studies and confirmed this result (Figure S1B). The higher I^2^ (mITT: 83.7%) and the Funnel plot (Figure S2B) point to a difference in study outcome, most likely influenced by differences in trial design and analyses.

#### Comparison of INI versus placebo both in combination with optimized background

In the BENCHMRK 1 and 2 trials comparing raltegravir (n = 459) to placebo (n = 237) in patients experiencing therapy failure, superior and sustained viral suppression was observed up to 96 weeks (mITT, 24 weeks: 62% versus 33% virological suppression, p<0.001) [2], [35]. The majority of virus isolates of the raltegravir failure patients harbored integrase resistance mutations, most of which were already detected by 24 weeks of therapy. In the Protocol 005 study, the efficacy of several raltegravir dosages (200 mg, 400 mg or 600 mg bd) (n = 134) with optimized background versus placebo (n = 45) were evaluated in highly experienced patients with HIV RNA >5000 copies/ml [36], [37]. At 24 weeks, 62.0% in all raltegravir treated groups versus 11.3% in the placebo group reached an undetectable viral load (mITT). Integrase mutations were observed in 35/38 INI treated patients failing therapy.

#### Comparison of INI versus PI both in combination with optimized background

In the GS-183-105 dose ranging study, elvitegravir plus optimized background regimen was compared with ritonavir boosted PI plus optimized background regimen (n = 63) in predominantly high PI experienced patients [38]. In the 50 mg qd arm (n = 75) elvitegravir was non-inferior while in the 125 mg arm (n = 73) elvitegravir was superior at reaching successful virological outcomes after 24 weeks (mITT time-weighted average change in log10 HIV-1 RNA (DAVG) treatment difference: −0.42, 95% CI −0.77 to −0.06, p = 0.021). This time-weighted endpoint has not been further validated in other clinical trials. No OT and AT data could be extracted for this study.

A sub-set of additional trials were reviewed but could not be included in the meta-analysis for various reasons. Three uncontrolled trials evaluated raltegravir in treatment-experienced patients in combination with other relatively new compounds. The first study is an open-label study describing the use of raltegravir with etravirine (400 mg bid) and an optimized background in patients experiencing treatment failure with darunavir (n = 20) [39]. 65% of participating patients obtained viral suppression at week 24 (mITT). The second study is ANRS 139 TRIO, which combined raltegravir and etravirine with ritonavir boosted darunavir (n = 103) in highly treatment-experienced patients experiencing virological failure. In this landmark study, the combination of three new compounds resulted in - at that time - fascinating high virological suppression rates (mITT: 86% at 48 weeks) among treatment-experienced patients and these results persisted up to 96 weeks [3], [40]. Lastly, raltegravir in combination with etravirine and maraviroc (n = 28) was tested in an uncontrolled study among patients experiencing therapy failure. This approach resulted in high levels of virological suppression (mITT: % of patients HIV RNA <50 copies/ml: 92% at 48 weeks) and no virological failure [41].

Functional mono-therapy with raltegravir in triple-resistant patients (n = 67) was evaluated in one observational trial and showed high efficacy at 48 weeks (mITT: 64% of patients with HIV RNA <50 copies/ml) [42]. Raltegravir resistance was observed in all patients experiencing virological failure.

The GS-183-0145 study compared once-daily elvitegravir versus twice-daily raltegravir in combination with a fully active ritonavir boosted PI and a second agent in patients with virological failure [43]. This study could not be included in the meta-analysis because of comparison of two INI. Elvitegravir was non-inferior to raltegravir regarding virological response at 48 weeks (mITT). In case of failure, HIV-1 integrase resistance patterns by both drugs were comparable, indicating a similar genetic barrier and cross-resistance between both drugs [44].

The VIKING trials evaluating dolutegravir in raltegravir treatment-experienced patients could not be included in the meta-analysis due to their uncontrolled design [45]–[47]. In VIKING I dolutegravir 50 mg once daily (n = 27) and in VIKING II dolutegravir 50 mg twice daily (n = 24) were applied as functional mono-therapy for 10 days, followed by a subsequent replacement of the failing regimen by an optimized backbone. Use of dolutegravir in these cohorts resulted in a very high level of virological suppression at day 11 (mITT HIV RNA <50 copies/ml: 78% respectively 96%). Persistent viral suppression was observed in 41% respectively 52% of these highly experienced patients at week 24. On 15 paired viral isolates from day 1 and day 11 of VIKING II, 3/15 patients harboured additional raltegravir associated mutations. It could not be distinguished whether those mutations had been selected during initial raltegravir treatment or de novo during subsequent dolutegravir use [47].

### Clinical outcome in treatment-experienced patients with suppressed viral load

Three studies on ART-experienced patients switching to INI while virologically suppressed were included in the meta-analysis: the analysis based on mITT data indicated a non-significant unfavourable OR when an antiretroviral drug was switched to an INI (OR 1.43; 95% CI 0.89–2.31) (Figure 3C). The meta-analysis based on AT data (OR 1.73, 95% CI 1.01–2.97) demonstrates inferiority after such switch (Figure S1C).

#### Comparison of INI versus PI both in combination with dual NRTI

In the SWITCHMRK 1 and 2 studies (n = 347 both arms), a switch from ritonavir boosted lopinavir towards raltegravir based ART was evaluated compared to continuation of the lopinavir based therapy [48]. Baseline genotyping was not performed as patients had an undetectable viral load at screening. Archived resistance data were not taken into account. The studies were terminated prematurely because non-inferiority of raltegravir to ritonavir boosted lopinavir was not established at week 24 (mITT: treatment difference −6.2%, 95% CI −11.2 to −1.3). The lower success rate in the raltegravir arm was most likely due to inclusion of patients with a history of therapy failure and pre-existing resistance against the NRTI backbone. Patients without previous virological failure had similar virological response rates at week 24 in both arms. The majority of the assessable patients who rebounded on raltegravir-based therapy harbored raltegravir resistant virus.

In SPIRAL, a second open-label trial, a switch of ritonavir boosted PI towards raltegravir (n = 139) versus continuation of the ritonavir boosted PI (n = 134) was evaluated in patients with well documented treatment history and long-term virological suppression [49]. In the mITT analysis at week 32, the switch from any ritonavir boosted PI to raltegravir in patients with undetectable viral load resulted in comparable high rates of virological suppression. Low-level raltegravir resistance was observed in only one patient.

#### Comparison of INI versus enfuvirtide both with background regimen

In the EASIER-ANRS 138 trial two switch strategies were compared: one group switched immediately to raltegravir (n = 85), the second group continued the low genetic barrier drug enfuvirtide and switched only after 24 weeks (n = 85). When analyzing the mITT 24 week data, the switch from enfuvirtide to raltegravir in heavily pretreated patients with a viral load <400 copies/ml at inclusion, resulted in similar rates of viral suppression [50]. No raltegravir resistance was detected upon virological failure.

Four smaller observational single-armed studies – hence not include in the meta-analysis - evaluated the switch from enfuvirtide to raltegravir in patients with an undetectable viral load and reported high virological success rates at weeks 16 to 48 [51]–[54].

The ODIS trial evaluated two dosage schemes of raltegravir – not included in the meta-analysis - while switching from a protease inhibitor and found that the 800 mg once daily arm (n = 149) had higher rates of virological failure at 24 weeks compared to 400 mg twice-daily (n = 73). In patients with prior NRTI resistance, significant higher failure rates were seen in both arms [55]. RASTA (Raltegravir Simplification for Toxicity or Adverse events) compared switching to raltegravir 400 mg either with tenofovir/emtricitabine (n = 21) or with abacavir/lamuvidine (n = 19) in patients on PI, NNRTI or NRTI-based therapy with suppressed viral load and found comparable virological suppression rates at 24 weeks. Only one patient experienced therapy failure after switch [56]. Anecdotal data from another small study (n = 20) which could not be included in the meta-analysis, showed high virological suppression up to 48 weeks in 96% of patients following regimen simplification towards a low genetic barrier regimen with raltegravir plus nevirapine (n = 20). Prior to the simplification, these patients were long term suppressed on a regimen containing nevirapine most likely without a history of therapy failure [57].

Although several studies have been performed investigating the intensification effect of adding an INI to a successful regimen, the body of evidence from those studies is graded as insufficient [58]–[66]. The heterogeneous nature of the studies, using different outcome measures to assess clinical outcome, residual immune activation and viral replication, and the duration of intensification makes comparison and inclusion in a meta-analysis impossible.

Five other uncontrolled studies describing a switch to raltegravir and boosted or unboosted PI reported good results but the evidence graded as insufficient [67]–[71]. A varying percentage of participants with an undetectable viral load at start of those studies (63% to 100%), different outcome measures and study duration, all hampered uniform conclusions.

### Pooled analysis of immunological efficacy, adverse events and emergence of drug resistance

When assessing the immunological response after start of INIs, the majority of the controlled studies with raltegravir, elvitegravir or dolutegravir indicate a similar median CD4 increase compared to other regimens. However, in therapy-naive patients, GS-236-0102 (48w), SINGLE (48w) and the long-term follow-up of STARTMRK (240w), all reported significantly higher CD4 increments compared to efavirenz-based therapies [14], [19], [25]. In the subgroup of treatment-experienced patients with virological failure, use of raltegravir resulted in significant better immunological outcome in BENCHMRK 1 and 2 compared to placebo (96w) [35]. ODIS reported similar significant results after switching to raltegravir from a boosted PI (24w) [55].

The INIs are generally well tolerated and rarely Grade 3 or 4 treatment-emerging adverse events are reported. Compared to efavirenz, discontinuation from INIs due to clinical adverse events is infrequent, while compared to PIs, less severe and life-threatening laboratory abnormalities are observed. An overview of the major adverse events of all INIs can be found in Table 1.

In case of treatment failure in therapy-naive patients, few but high-level raltegravir and elvitegravir resistance was observed, which often conferred cross-resistance to these drugs. No resistance for dolutegravir in this patient population was detected. When combined with dual NRTI, the occurrence of raltegravir or elvitegravir resistance-associated mutations (RAM) was associated in 50% of cases with resistance to NRTI (Table S1).

## Discussion

We performed a systematic review on all published clinical data concerning integrase inhibitors and subsequently meta-analyses on the virological outcome of those studies which included a controlled arm. Based on the meta-analyses, treatment with INIs in combination with dual NRTI showed to be more beneficial for treatment-naive patients compared to other currently used treatment strategies. Also in treatment-experienced patients with virological failure, use of INIs proved to be beneficial as well. However, in successfully treated patients with a history of therapy failure, switching a high genetic barrier drug towards an INI was not supported. More in depth, the following indications for use of integrase inhibitors can be summarized:

### Initial therapy

The meta-analysis shows a significant OR in favor of INI combined with dual NRTI based on mITT and OT data, with a similar favorable trend when AT data are used. As both mITT and OT based meta-analyses show a similar significant OR, the clinical benefit of INIs is not only driven by improved tolerability, but also by higher antiviral efficacy. The non-significance of the AT-based meta-analysis can be due to small differences between OT and AT study populations, or might be influenced by the non-availability of AT data from a large dolutegravir trial.

In recent European and US treatment guidelines, raltegravir with a tenofovir/emtricitabine backbone is listed among the preferred regimens for antiretroviral-naive HIV infected individuals [4], [5], [72]. This is supported by the meta-analyses. Raltegravir showed comparable high virological efficacy compared to efavirenz as first line antiretroviral regimen, but was found to be superior driven by its good toxicity profile and tolerability [17], [18]. Besides its good tolerability, raltegravir has a limited risk for drug-drug interactions [73], [74]. Disadvantages of raltegravir are the non-availability of a single tablet regimen and the twice-daily dosing schedule, as supported by the QDMRK study [23]. Raltegravir showed a low genetic barrier to drug resistance upon failure. The emergence of raltegravir resistance was infrequent, but often of high-level (at least two INI RAMs) and transferring cross-resistance to elvitegravir, confirming resistance profiles observed in earlier vitro studies [75].

More recently developed INIs like elvitegravir and dolutegravir hold promise as part of a single tablet regimen (STR) in first-line therapy. Boosted elvitegravir as part of a STR revealed promising results in two large trials, but caution is needed because of increased INI and NRTI resistance. A similar low genetic barrier to drug resistance upon failure was seen for elvitegravir. Raltegravir and elvitegravir based regimens showed comparable or superior immunological response compared to other regimens. Dolutegravir combined with abacavir/lamuvidine has been the first combination reported to be virologically and immunologically superior compared to an efavirenz-based regimen. No drug resistance was detected suggesting a high genetic barrier to resistance development.

In this patient population, novel treatment strategies have been explored, such as the combination of INI with a PI, sparing the NRTIs. Individual studies are underpowered or failed to show superiority. Also the mITT, OT and AT-based meta-analysis failed to show significant odds ratios in favor of these nucleoside-sparing regimens. For stronger conclusions, more data are needed. Currently a large trial evaluating this concept (NEAT-001, tenofovir/emtricitabine/boosted darunavir versus raltegravir/boosted darunavir) is underway.

### Virological failure

The meta-analyses demonstrate convincing evidence for a treatment change towards a regimen containing raltegravir or elvitegravir compared to placebo and PI in PI pre-treated individuals with virological failure, based on mITT as well as OT and AT data. For dolutegravir, no randomized trials were available to include in the meta-analysis. Cross-resistance observed in these studies suggests no additional value for sequential use of raltegravir and elvitegravir. In contrast, dolutegravir has limited cross-resistance to other INIs based on a recent report, which could infer a potential role for this drug in INI-experienced patients depending on the resistance profile [45]–[47], [76], [77]. A timely switch or even interruption of raltegravir or elvitegravir may prevent accumulation of resistance and should be considered in order to maximize the potential effect of dolutegravir [78]. Similar or superior immunological response was observed for raltegravir and elvitegravir based regimens compared to other regimens.

### Regimen simplification

Switching from enfuvirtide to raltegravir resulted in high levels of durable suppression in several uncontrolled trials [51]–[54] indicating that substitution of a low genetic barrier component of combination antiretroviral therapy by raltegravir in patients with documented or suspected drug resistance can be safely performed [50]. In contrast, the switch from a high genetic barrier PI towards raltegravir in a similar population resulted in a unfavorable OR in the OT-based meta-analysis, and thus higher levels of therapy failure in the raltegravir arm. When adding the effect of adherence and tolerability (mITT), the unfavorable effect was less evident. Two major studies revealed conflicting results possibly influenced by duration of suppression and documentation of treatment history [48], [49]. The results indicate that when switching virologically suppressed patients, individual patient management is needed to assess history of treatment failure, available resistance profiles and duration of the current suppressive regimens in order to perform a safe switch.

### Limitations of the systematic review and meta-analysis

One of the limitations of this systematic review and meta-analysis are potential variations in efficacy between the individual INIs compared in similar settings, inherent to the study methodology. Furthermore, the virological outcome data were obtained following different protocols. However, direct comparison of these methodologies has not revealed major differences in outcome [79]–[81]. The SINGLE trial could not be incorporated in the AT meta-analysis for therapy-naive patients, since the number of patients failing combination antiretroviral therapy during the study were not reported so far. In reviewing the current literature on the clinical use of INIs, it becomes obvious that certain clinical questions cannot be answered because of insufficient evidence due to the lack of controlled studies. One of these gaps concerns effect of treatment intensification. Another gap concerns use of INI during pregnancy, since good tolerability and rapid decline of HIV RNA in the plasma suggests a place for integrase inhibitors in this setting [82]. However, there is no evidence from large trials on efficacy and teratogenicity. Similarly, insufficient data are available to include raltegravir in standard post exposure prophylaxis regimens. With the anticipated arrival of studies with new available INIs these gaps could be closed. Finally, the review was restricted to English-language reports.

## Conclusion

The meta-analyses positioned INI as a preferred drug in the setting of treatment-naive and as beneficial addition in treatment-experienced patients with virological failure, based on virological efficacy. Careful use of INI when replacing a high genetic barrier PI is warranted. The perspectives of new single tablet regimens containing elvitegravir or dolutegravir taken in the absence of food restrictions hold promise for broad use in first line regimens. In general, the addition of the integrase inhibitor class to our armamentarium has strengthened cART regimens, and further rational use can preserve future therapeutic options.

## Supporting Information

Figure S1 **Forest Plots of OT/AT meta-analyses.** Panel A: Forest plot showing the meta-analysis of OT and AT data extracted from studies with therapy-naïve patients. Panel B: Forest plot showing the meta-analysis of OT and AT data extracted from studies with ART-experienced patients in case of virological failure. Panel C: Forest plot showing the meta-analysis of OT and AT data extracted from studies with ART-experienced patients switching with suppressed viral load. OT = on-treatment; AT = as-treated; ART = antiretroviral treatment; INI = integrase inhibitor; (N)NRTI = (non-)nucleoside reverse transcriptase inhibitor; PI = protease inhibitor; T20 = enfuvirtide: OR = odds ratio.
(TIF)

Figure S2 **Funnel Plots of the mITT meta-analyses.** A funnel plot is a scatterplot of treatment effect against a measure of study size. It is used as an aid to detect bias or systematic heterogeneity. A symmetric inverted funnel shape arises from a ‘well-behaved’ data set, in which bias is unlikely while an asymmetric funnel indicates a relationship between treatment effect and study size. The three funnel plots shown for this systematic review and meta-analyses are based on mITT data and are all symmetric. Panel A: Funnel plot for the meta-analysis of mITT data extracted from studies with therapy-naïve patients. Panel B: Funnel plot for the meta-analysis of mITT data extracted from studies with ART-experienced patients in case of virological failure. Larger mathematical differences, small number of studies and small population size in some studies may skew the plot. Panel C: Funnel plot for the meta-analysis of mITT data extracted from studies with ART-experienced patients switching with suppressed viral load. mITT = modified intention-to-treat.
(TIF)

Table S1 **Overview of resistance data in the controlled studies on INI use.** Of the controlled studies on INI use in clinical settings, data were extracted on emergence of drug resistance. The endpoint of data-extraction, as well as the population size in the INI-arm and control arm are reported, besides the number of patients experiencing treatment failure in each arm. For each drug class, the proportion of patients harboring viruses with resistance-associated mutations is indicated in relation to the evaluable patient samples. INI = integrase inhibitor; CTR = control arm; (w) = weeks; VF = virological failure; RAL = raltegravir; EFV = efavirenz; EVG = elvitegravir; DTG = dolutegravir; (N)NRTI = (non-) nucleoside reverse transcriptase inhibitor; PI = protease inhibitor; RAM = resistance-associated mutation.
(DOCX)
