# Supplementary figures and images for: Clinical Use of HIV Integrase Inhibitors: A Systematic Review and Meta-Analysis
Source: PLoS One. 2013 Jan 9;8(1):e52562. doi: 10.1371/journal.pone.0052562 (PMC3541389; doi:10.1371/journal.pone.0052562)

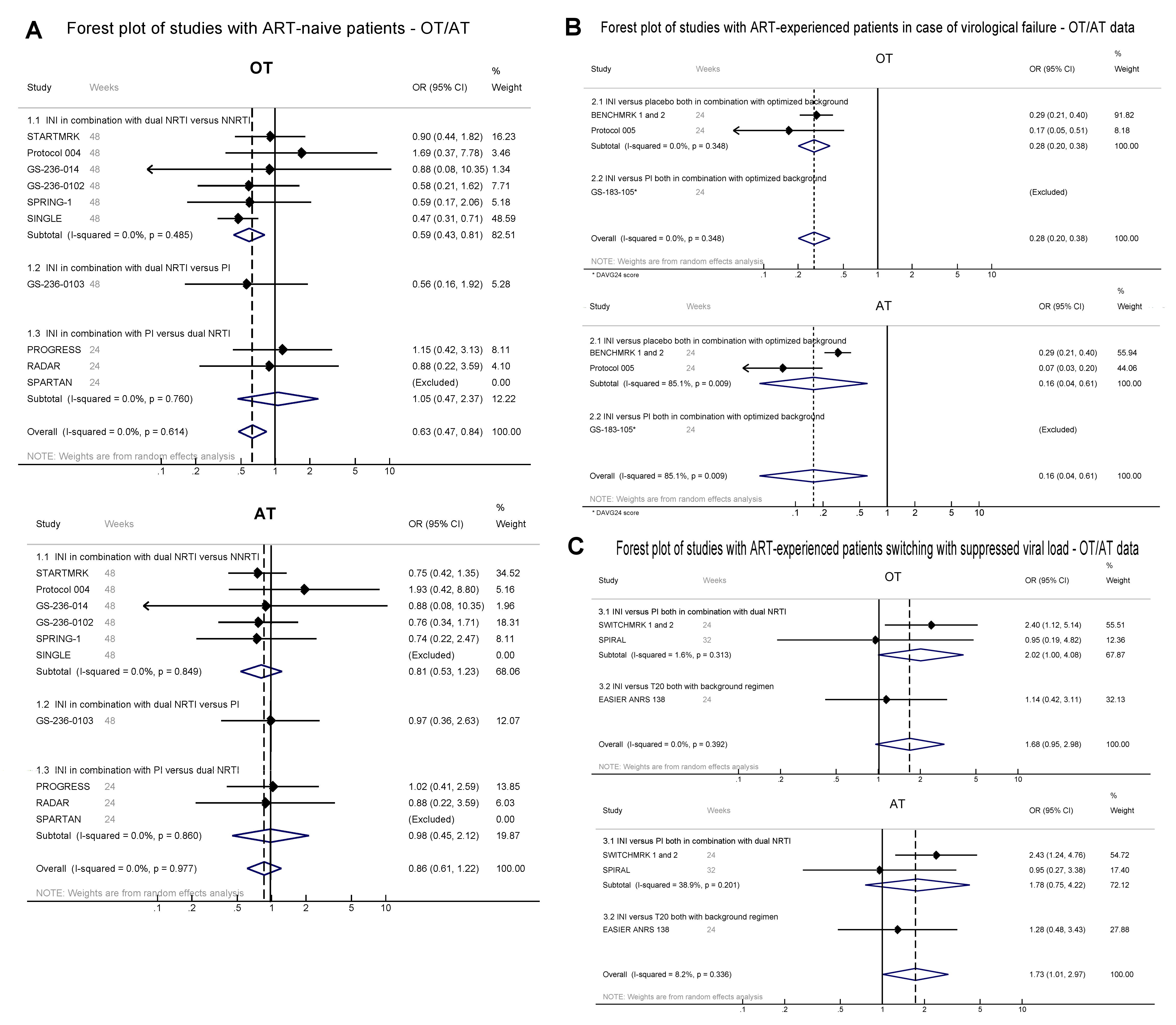

Supplement: Figure S1 — Forest Plots of OT/AT meta-analyses. Panel A: Forest plot showing the meta-analysis of OT and AT data extracted from studies with therapy-naïve patients. Panel B: Forest plot showing the meta-analysis of OT and AT data extracted from studies with ART-experienced patients in case of virological failure. Panel C: Forest plot showing the meta-analysis of OT and AT data extracted from studies with ART-experienced patients switching with suppressed viral load. OT = on-treatment; AT = as-treated; ART = antiretroviral treatment; INI = integrase inhibitor; (N)NRTI = (non-)nucleoside reverse transcriptase inhibitor; PI = protease inhibitor; T20 = enfuvirtide: OR = odds ratio. (TIF) [file pone.0052562.s001.tif]

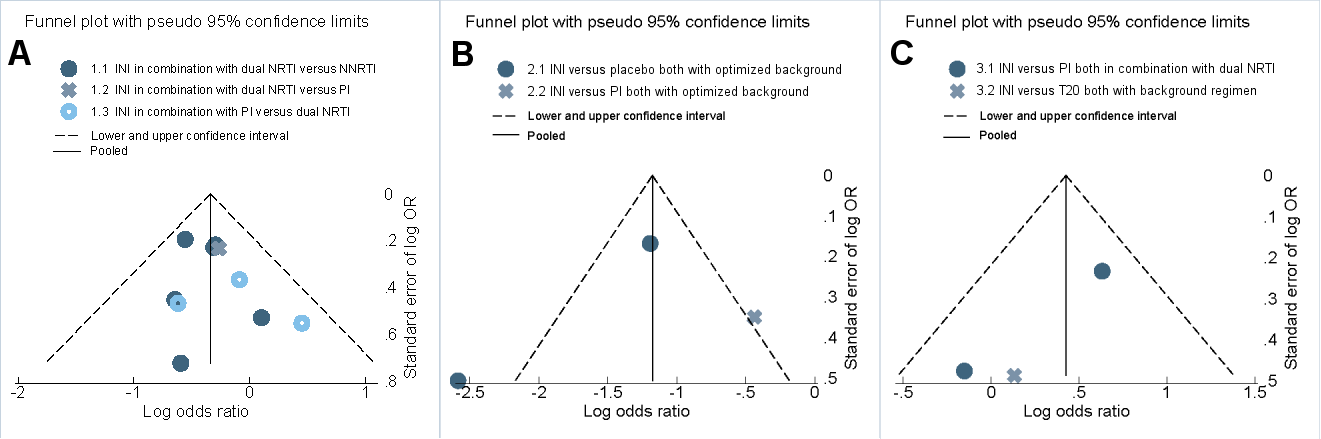

Supplement: Figure S2 — Funnel Plots of the mITT meta-analyses. A funnel plot is a scatterplot of treatment effect against a measure of study size. It is used as an aid to detect bias or systematic heterogeneity. A symmetric inverted funnel shape arises from a ‘well-behaved’ data set, in which bias is unlikely while an asymmetric funnel indicates a relationship between treatment effect and study size. The three funnel plots shown for this systematic review and meta-analyses are based on mITT data and are all symmetric. Panel A: Funnel plot for the meta-analysis of mITT data extracted from studies with therapy-naïve patients. Panel B: Funnel plot for the meta-analysis of mITT data extracted from studies with ART-experienced patients in case of virological failure. Larger mathematical differences, small number of studies and small population size in some studies may skew the plot. Panel C: Funnel plot for the meta-analysis of mITT data extracted from studies with ART-experienced patients switching with suppressed viral load. mITT = modified intention-to-treat. (TIF) [file pone.0052562.s002.tif]
