# Supplementary material for: Clinical Use of HIV Integrase Inhibitors: A Systematic Review and Meta-Analysis
Source: PLoS One. 2013 Jan 9;8(1):e52562. doi: 10.1371/journal.pone.0052562 (PMC3541389; doi:10.1371/journal.pone.0052562)
Supplement: Table S1 — Overview of resistance data in the controlled studies on INI use. Of the controlled studies on INI use in clinical settings, data were extracted on emergence of drug resistance. The endpoint of data-extraction, as well as the population size in the INI-arm and control arm are reported, besides the number of patients experiencing treatment failure in each arm. For each drug class, the proportion of patients harboring viruses with resistance-associated mutations is indicated in relation to the evaluable patient samples. INI = integrase inhibitor; CTR = control arm; (w) = weeks; VF = virological failure; RAL = raltegravir; EFV = efavirenz; EVG = elvitegravir; DTG = dolutegravir; (N)NRTI = (non-) nucleoside reverse transcriptase inhibitor; PI = protease inhibitor; RAM = resistance-associated mutation. (DOCX) [file pone.0052562.s003.docx]

|  | **Endpoint (w)** | **INI (n)** | **Ctr (n)** | **VF – INI (n)** | **VF – ctrl (n)** | **Resistance in INI arm** | **Resistance in ctrl arm** |
| --- | --- | --- | --- | --- | --- | --- | --- |
| **STARTMRK** | *156* | 281 | 282 | 50 | 54 | 4/19 RAL RAMs; 6/19 FTC RAMs | 7/16 EFV RAMs; 5/16 FTC RAMs |
| **Protocol 004** | *96* | 160 | 38 | 6 | 2 | 3/6 RAL RAMSs; 1/6 TDF RAMs; 4/6 3TC RAMs | 2/2 EFV RAMs; 2/2 FTC RAMs |
| **GS-236-0102** | *48* | 348 | 352 | 14 | 17 | 7/14 EVG RAMs; 8/14 NRTI RAMs; 0/14 NNRTI RAMs | 8/17 EFV RAMs; 2/17 NRTI RAMs; 0/17 INI RAMs |
| **SPRING-1** | *48* | 155 | 50 | 10 | 4 | 0/2 DTG RAMs; 0/2 INI RAMs; 1/2 NRTI RAMs | 0/1 EFV RAMs; 0/1 NRTI RAMs; 0/1 INI RAMs |
| **GS-236-0103** | *48* | 353 | 355 | 12 | 8 | 4/12 EVG RAMs; 4/12 NRTI RAMs | 0/8 PI RAMs; 0/8 NRTI RAMs; 0/8 INI RAMs |
| **SPRING -2** | *48* | 413 | 414 | 21 | 33 | No treatment-emergent resistance was observed | 1/? RAL RAMs; 4/? NRTI RAMs |
| **QDMRK** | *48* | 382 | 388 | 67 | 39 | 9/27 RAL RAMs; 11/28 FTC RAMs | 2/12 RAL RAMs; 4/13 FTC RAMs |
| **SPARTAN** | *24* | 63 | 31 | 11 | 8 | 4/5 RAL RAMs; 0/6 PI RAMs | No treatment-emergent resistance was observed |
| **PROGRESS** | *48* | 101 | 105 | 10 | 10 | 2/4 RAL RAMs; 0/4 PI RAMs | 1/3 FTC RAMs; 0/3 TDF RAMs; 0/3 PI RAMs |
| **RADAR** | *24* | 40 | 40 | 4 | 5 | No treatment-emergent resistance was observed | No treatment-emergent resistance was observed |
| **BENCHMRK 1 and 2** | *96* | 462 | 237 | 150 | 148 | 73/112 RAL RAMs | No data |
| **Protocol 005** | *24* | 133 | 45 | 38 | 38 | 35/38 RAL RAMs | No data |
| **GS-183-0145** | *48* | 205 | 73 | 70 | 77 | 16/60 EVG new RAMs; 4/59 PI RAMs; 8/59 NNRTI RAMs; 7/59 NRTI RAMs | 15/72 RAL new RAMs; 3/75 PI RAMs; 5/75 NNRTI RAMs; 10/75 NRTI RAMs |
| **SWITCHMRK 1 and 2** | *24* | 353 | 354 | 32 | 17 | 9/11 RAL RAMs; 6/11 NRTI RAMs | 1/3 RAL RAMs; 1/3 NRTI RAMs |
| **SPIRAL** | *48* | 139 | 134 | 4 | 6 | 0/1 RAL RAMs; 0/1 PI or NRTI RAMs | 3/4 extensive PI or NRTI RAMs |
| **ODIS** | *24* | 149 | 73 | 12 | 1 | 8/13 RAL RAM; 10/13 NRTI RAM | |

**Supplementary Table S1: Overview of resistance data in the controlled studies on INI use**
